# Test-retest reliability and convergent validity of (R)-[^11^C]PK11195 outcome measures without arterial input function

**DOI:** 10.1186/s13550-018-0455-8

**Published:** 2018-11-29

**Authors:** Pontus Plavén-Sigray, Granville James Matheson, Zsolt Cselényi, Aurelija Jucaite, Lars Farde, Simon Cervenka

**Affiliations:** 1 Department of Clinical Neuroscience, Center for Psychiatry Research, Karolinska Institutet and Stockholm County Council, SE-171 76 Stockholm, Sweden; 2 PET Imaging Centre, Precision Medicine and Genomics, IMED Biotech Unit, AstraZeneca, Karolinska Institutet, Stockholm, Sweden

**Keywords:** (R)-[^11^C]PK11195, Arterial input function, Reference region, Supervised cluster analysis, Test-retest, Reliability

## Abstract

**Purpose:**

The PET radioligand (R)-[^11^C]PK11195 is used to quantify the 18-kDa translocator protein (TSPO), a marker for glial activation. Since there is no brain region devoid of TSPO, an arterial input function (AIF) is ideally required for quantification of binding. However, obtaining an AIF is experimentally demanding, is sometimes uncomfortable for participants, and can introduce additional measurement error during quantification. The objective of this study was to perform an evaluation of the test-retest reliability and convergent validity of techniques used for quantifying (R)-[^11^C]PK11195 binding without an AIF in clinical studies.

**Methods:**

Data from six healthy individuals who participated in two PET examinations, 6 weeks apart, were analyzed. Regional non-displaceable binding potential (BP_ND_) values were calculated using the simplified reference tissue model, with either cerebellum as reference region or a reference input derived using supervised cluster analysis (SVCA). Standardized uptake values (SUVs) were estimated for the time interval of 40–60 min.

**Results:**

Test-retest reliability for BP_ND_ estimates were poor (80% of ICCs < 0.5). BP_ND_ estimates derived without an AIF were not correlated with BP_ND_, total or specific distribution volume from the 2TCM using an AIF (all *R*^2^ < 12%). SUVs showed moderate reliability but no correlation to any other outcome measure.

**Conclusions:**

Caution is warranted when interpreting patient-control comparisons employing (R)-[^11^C]PK11195 outcome measures obtained without an AIF.

**Electronic supplementary material:**

The online version of this article (10.1186/s13550-018-0455-8) contains supplementary material, which is available to authorized users.

## Introduction

(R)-[^11^C]PK11195 was the first positron emission tomography (PET) radioligand developed for quantification of the translocator protein (TSPO). Within the brain, TSPO is mainly expressed in glial cells. Based on in vitro studies showing increases in TSPO expression in response to pro-inflammatory stimuli, the protein has been considered a biomarker for brain immune activation [1]. As such, (R)-[^11^C]PK11195 has, since the early 1990s, been applied in a wide range of clinical studies [2].

TSPO is expressed throughout the brain which means that no region can serve as a reference for quantification of specific (R)-[^11^C]PK11195 binding. Instead, a metabolite-corrected arterial input function (AIF) must be obtained and used as an input function for a kinetic model from which binding parameters can be estimated. Common measures of regional binding derived from the use of an AIF are total distribution volume (*V*_T_), specific distribution volume (*V*_S_ or BP_P_), and binding potential (BP_ND_) [3].

Obtaining a metabolite-corrected input function is costly, often uncomfortable for research participants, and can also be prone to measurement error. Therefore, alternative approaches for quantifying binding have been suggested which are less demanding and which do not require an AIF. The most simple method is to calculate the radioactivity concentration in a brain region normalized by the injected radioactivity and the subject’s weight (standardized uptake value or SUV). As such, the SUV does not directly reflect specific binding since the signal also contains non-specific binding and radioactivity from vasculature. Importantly, SUVs are also dependent on the rate and extent of radioligand delivery to the brain. This means that results may be influenced by cerebral blood flow or peripheral changes such as differences in metabolism or blood binding. For TSPO, previous studies have shown a high correlation between (R)-[11C]PK11195 *V*_T_ and SUVs in synovial tissue in the knee joint [4, 5]. However, it has been shown that in pathological conditions characterized by reduced rates of flow or variations in blood volume, the use of SUVs for quantifying (R)-[11C]PK11195 brain uptake yields biased and unprecise outcomes [6]. In addition, the high concentration of TSPO in peripheral tissues, which may be altered during peripheral inflammation, can greatly influence the extent of radioligand brain delivery [7]. Due to these reasons, SUVs are unlikely to be an unbiased index of TSPO binding in brain.

An alternative method for quantifying (R)-[^11^C]PK11195 binding without the use of an AIF is the supervised cluster analysis (SVCA) method [8, 9]. SVCA, which is performed on dynamic PET images, aims to segment voxels into classes, differentiated by their kinetic behavior. The goal is to isolate gray matter (GM) voxels assumed to contain negligible levels of specific binding. These voxels are then used to establish a time-activity curve (TAC) serving as a reference input in a kinetic model, such as the simplified reference tissue model (SRTM) [10]. However, application of the SVCA method has some limitations. The kinetic classes can produce different results depending on scanner type, acquisition protocol, and radioligand, and it is important to evaluate the classes prior to applying them clinically. The SVCA method has been used in (R)-[^11^C]PK11195 studies to compare TSPO binding between healthy control subjects and patients with Alzheimer’s disease [11, 12], multiple sclerosis [13], traumatic brain injury [14], and schizophrenia [15] or studies which examined changes in TSPO expression in normal aging [16, 17].

Another simplified approach to obtain BP_ND_ values without arterial sampling is to use a reference tissue model with the cerebellum as reference region, despite the fact that the cerebellum contains non-negligible levels of TSPO [18]. Since there is specific binding of (R)-[^11^C]PK11195 in the reference region, ensuing BP_ND_ values will not reflect the “true” binding potential but rather pseudo-binding potential (pseudo-BP_ND_). One previous study found that pseudo-BP_ND_ was correlated to BP_ND_ from a constrained version of the 2TCM, using a sample consisting of elderly and patients with traumatic brain injury [19]. Pseudo-BP_ND_ values have also been used, for example, to compare (R)-[^11^C]PK11195 binding in healthy controls and in patients with schizophrenia [20, 21], major depressive disorder [22], and glioma [23].

In order for PET quantification methods to be useful in clinical studies, they should yield outcomes which are both reliable and valid. Two previous studies evaluated a set of different methods, with and without AIF, for quantifying (R)-[^11^C]PK11195 binding [19, 24], but did not examine the test-retest reliability of ensuing outcome measures. In a previous test-retest study of six healthy subjects performed at our center, the reliability of (R)-[^11^C]PK11195 BP_ND_ values obtained using AIF were found to be very poor in most target regions examined [25]. In contrast, the test-retest reliability of (R)-[^11^C]PK11195 BP_ND_ using SRTM with SVCA reference has been evaluated in four patients with Alzheimer’s disease [8]. In that study, ICC values were found to be high in most regions of interest. However, no study has yet examined the test-retest reliability of SVCA in healthy controls. To our knowledge, the reliability of (R)-[^11^C]PK11195 SUV or BP_ND_ from SRTM with the cerebellum as reference has never been reported in healthy control subjects, despite both outcomes being applied in clinical patient-control comparisons.

The main objective of this study was to evaluate the test-retest reliability and repeatability of (R)-[^11^C]PK11195 (1) SUVs and (2) BP_ND_ obtained from SRTM, using cerebellum or SVCA-derived voxels as reference, respectively. The second objective was to examine the convergent validity of these outcomes by correlating them to *V*_T_, *V*_S_, and BP_ND_ values derived using an AIF.

## Methods and materials

### Subjects and imaging procedures

In the present analysis, we included PET examinations from six healthy male subjects (mean age = 25.8±3.9) who participated in a previous test-retest study of (R)-[^11^C]PK11195 [25]. All subjects gave written informed consent according to the Helsinki Declaration prior to their participation in the original study. The study was approved by the Karolinska University Hospital Radiation Safety Committee and the Regional Ethics Committee in Stockholm.

All subjects participated in two PET measurements that took place approximately 6 weeks apart and were run on an ECAT Exact HR 47 system (Siemens/CTI, Knoxville, TN, USA). Structural magnetic resonance imaging (MRI) examinations were performed on a Siemens 1.5T Magnetom, resulting in a T1-weighted image for each subject. Production and radio-synthesis of (R)-[^11^C]PK11195 has been described previously [25]. Mean injected radioactivity was 302 ± 33 MBq. Arterial samples were obtained in all PET measurements, from which a metabolite-corrected AIF was derived (see [25]).

ROI delineation was performed on the subjects’ T1-weighted images using the FreeSurfer software (5.0.0, http://surfer.nmr.mgh.harvard.edu/). ROIs were co-registered to PET images using SPM5 (Wellcome Department of Cognitive Neurology, UK). Sixty-three-minute TACs were extracted for the whole of GM, frontal cortex, striatum, thalamus, hippocampus, and cerebellum (CER), except for one PET examination where only a 50-min scan was obtained.

### Quantification of outcomes with and without AIF

The two-tissue compartment model (2TCM) with AIF was used to estimate kinetic rate constants. The fraction of blood volume in target tissue (vB) and the delay between start of the AIF and the ROI TAC were fitted using the 2TCM applied on the entire GM TAC. These parameters were held constant for the remaining ROI fits. *V*_T_, *V*_S_, and BP_ND_ were then calculated using the rate constants. In addition to the above, we also evaluated the outcomes from the 2TCM when fitting vB separately for each ROI, as this has been suggested to yield less bias and better fit for BP_ND_ (*k*_3_/*k*_4_) estimates [24].

SUVs were calculated from the average radioactivity concentration in frames spanning from 40 to 60 min of the regional TACs and dividing by the injected radioactivity and the subject’s body weight. A time span of 40–60 min was chosen since this has previously shown to produce SUVs which were associated with *V*_T_ in knee joints in patients with rheumatoid arthritis [4]. However, we also evaluated three additional time intervals spanning from 10 to 30, 20 to 40, and 30 to 50 min.

The original SVCA method classifies PET voxels into six different tissue types associated with distinct kinetic profiles: (1) GM with high specific binding, (2) GM with low specific binding, (3) white matter, (4) soft tissue, (5) bone, and (6) blood. It has been shown that removal of bone and soft tissue, by using a MRI defined brain mask, prior to performing SVCA reduced variability of binding estimates and improved correlation to outcomes derived using an AIF [26]. We therefore applied this restricted SVCA method (SVCA4), using the MATLAB software “Super-PK” (Imperial Innovations, Imperial College London). The Super-PK software was modified in order to be compatible with the scanning protocol applied in this study. Specifically, a cubic Gaussian smoothing kernel (FWHM 4 mm) was applied to all PET images prior to the analysis, and the 30-s background frame present in the population-based kinetic classes was removed. A reference TAC was then obtained for each PET measurement consisting of GM voxels classified as being associated with low specific binding. SRTM (called SRTM-SVCA4 below) was applied to estimate BP_ND_ for all ROIs. In this study, the primary results from SVCA4 method is based on the kinetic classes from Turkheimer et al. [8]. However, we also evaluated the reliability of outcomes derived using two additional sets of population-based kinetic classes: one set from VU University Medical Center Amsterdam (VUMC) [9] and an unpublished set from the Turku PET Centre (TPC). This was done in order to examine the robustness of the SVCA4 method when using different population-based kinetic classes.

It has been shown that by using a version of SRTM that takes the radioactivity contribution from the vasculature into account, separation in (R)-[^11^C]PK11195 SVCA4 derived BP_ND_ between patients with AD and healthy controls can be improved [9, 27]. In addition to the SRTM algorithm, this model (called SRTMv) estimates and corrects for the fraction of blood volume in both target and reference TACs, by using an image-derived blood curve. Hence, we also evaluated the performance of SRTMv when using a reference curve derived from SVCA4 (SRTMv-SVCA4). Image-derived blood curves were obtained by extracting radioactivity from the entire scan from a region defined by the 10 voxels of highest intensity from the first minute of each examination, as described previously [27].

The SRTM with the cerebellum as pseudo-reference region (SRTM-CER) was also applied on all PET measurements and TACs to obtain BP_ND_ values for each ROI.

Finally, we also calculated *V*_T_, *V*_S_, and BP_ND_ values from the cerebellum and the SVCA4 reference TAC using the 2TCM with an AIF. This was done in order to both ascertain that the results were similar to previously published data and to evaluate the reliability and stability of the reference input.

### Statistical analyses

The test-retest reliability, repeatability, and precision were examined by calculation of the intraclass correlation coefficient (ICC), the percentage average absolute variability (AbsVar), and the standard error of measurement (SEM), respectively. Since AbsVar can scale with the additive magnitude of the outcome, this particular metric is not suitable for comparing different outcomes with different means. We therefore also report the test-retest metric minimum detectable difference (MD). MD is based on the precision of an outcome (SEM) and is an approximation of the size of a difference from one measurement to another measurement which would be needed to detect a “real” change (according to a 95% confidence interval; [28]). MD is reported as a percentage of the absolute mean of the outcome, in order to allow for comparison between different measures.

Convergent validity was examined by correlating all outcomes without an AIF to those derived using an AIF. Although the outcomes are derived in different ways, they all aim to estimate, directly or indirectly, the specific binding to target. If they are valid outcomes, they should therefore be correlated to one other. For instance, both *V*_S_ and BP_ND_ are defined as being proportional to the availability of target (∝ *B*_avail_/*K*_d_), and a lack of correlation would therefore imply that one or both of the outcomes are expressing a high degree of error or imprecision. Another reason for evaluating the correlations between outcomes is to inform the design of future meta-analyses. Outcomes which are only weakly associated with one another should likely not be entered into the same meta-analytic model, as this would violate a critical statistical model assumption and thereby yield uninterpretable estimates of effect size.

All kinetic modeling were performed using the R-package “kinfitr” (version 0.3.0, www.github.com/mathesong/kinfitr) together with “nls.multstart” [29]. All statistical analyses were carried out in R (v.3.3.2 “Sincere Pumpkin Patch”).

## Results

### Test-retest reliability of outcome measures derived with and without an AIF

Table 1 shows the mean, SD, and test-retest metrics for all outcomes. BP_ND_ values from SRTM using SVCA4 and the cerebellum as reference, and SRTMv using SVCA4 as reference were in the same range as described previously for healthy control subjects [8, 9]. There was a large difference in magnitude of BP_ND_ values derived with and without the use of an AIF. Regional BP_ND_ values from the 2TCM were on average 7 times higher than BP_ND_ from SRTM-SVCA4 and over 700 times higher than BP_ND_ from SRTM-CER. *V*_T_ values for the SVCA4 reference TACs were of a similar magnitude and range (mean = 0.74, SD = 0.18, range = 0.49 to 0.96) compared to previously published results [9].

**Table 1:** Mean values (for both PET examinations) and test-retest reliability, repeatability, and precision estimated using the intra-class correlation coefficient (ICC), average absolute variability in percentage (AbsVar), and standard error of measurement (SEM) of different outcome measures derived with or without AIF. The minimum detectable difference (MD) denotes the difference (expressed as a percentage of the mean) needed between two measurements for them to be significantly different from each other

Measure	Region	Mean	SD	ICC	AbsVar%	SEM	MD%
V_T_ (2TCM)	FC	0.72	0.16	0.73	15	0.08	32
	GM	0.7	0.17	0.78	15	0.08	31
	HIP	0.72	0.19	0.66	21	0.11	44
	STR	0.76	0.17	0.44	18	0.13	46
	THAL	0.77	0.22	0.69	21	0.12	43
V_S_ (2TCM)	FC	0.42	0.09	0.68	14	0.05	32
	GM	0.42	0.09	0.67	15	0.05	34
	HIP	0.45	0.1	0.35	21	0.08	51
	STR	0.44	0.1	0.23	23	0.09	58
	THAL	0.48	0.14	0.91	13	0.04	24
BP_ND_ (2TCM)	FC	1.49	0.33	0.65	18	0.2	37
	GM	1.62	0.4	0.31	29	0.33	56
	HIP	2.02	0.77	− 0.19	50	0.84	115
	STR	1.41	0.39	0.32	22	0.32	63
	THAL	1.79	0.67	− 0.11	39	0.71	110
BP_ND_ (SRTM-SVCA4)	FC	0.17	0.04	0.21	29	0.04	63
	GM	0.21	0.06	0.34	27	0.05	59
	HIP	0.17	0.09	− 0.39	83	0.1	160
	STR	0.21	0.09	− 0.12	59	0.09	120
	THAL	0.35	0.09	0.32	22	0.07	55
BP_ND_ (SRTMv-SVCA4)	FC	0.15	0.09	− 0.49	113	0.11	194
	GM	0.22	0.1	0.04	62	0.1	122
	HIP	0.17	0.08	− 0.84	75	0.11	180
	STR	0.2	0.1	0.79	38	0.04	60
	THAL	0.36	0.12	− 0.14	46	0.13	97
BP_ND_ (SRTM-CER)	FC	− 0.07	0.09	0.5	160	0.06	258
	GM	− 0.03	0.06	0.51	277	0.04	444
	HIP	0.01	0.08	0.19	181	0.07	1920
	STR	− 0.02	0.17	− 0.14	196	0.18	2963
	THAL	0.09	0.06	0.67	494	0.04	112
SUV 40–60 min	FC	10.31	3.72	0.89	19	1.26	34
	GM	10.44	3.55	0.87	19	1.3	34
	HIP	10.43	3.36	0.82	19	1.43	38
	STR	10.27	3.64	0.8	26	1.63	44
	THAL	11.36	3.81	0.84	20	1.55	38

*FC* frontal cortex, *GM* gray matter, *HIP* hippocampus, *STR* striatum, *THAL* thalamus

In the present analysis, SUVs, *V*_T_, and *V*_S_ had the highest reliability across all ROIs (median ICC_SUV_ = 0.84; median ICC_VT_ = 0.69; median ICC_VS_ = 0.67). BP_ND_ from SRTM and SRTMv with SVCA4 reference showed the lowest overall reliability (median ICC = 0.21 and − 0.14).

SUV, *V*_T_, and *V*_S_ showed on the smallest (and therefore best) minimum detectable difference (median MD_SUV_ = 38; median MD_VT_ = 43; median MD_VS_ = 34), while BP_ND_ from SRTM-CER showed the highest MD (median MD = 444).

### Test-retest reliability of binding in the cerebellum and the SVCA4-derived reference

**Fig. 1:**
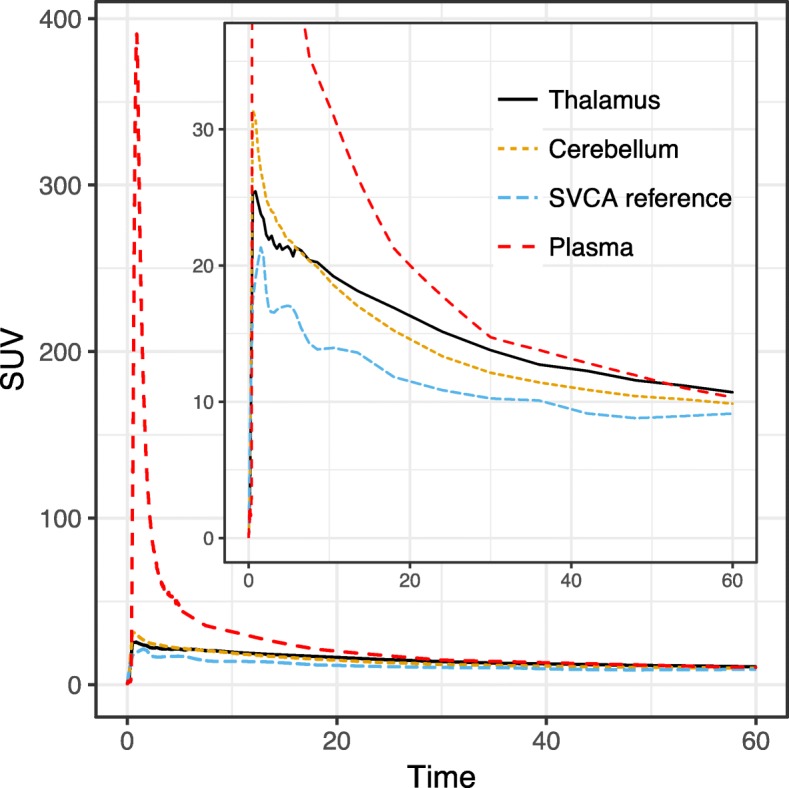
Average (R)-[^11^C]PK11195 reference (cerebellum and SVCA4) or input (metabolite-corrected plasma) TACs expressed in SUVs. The average thalamus TAC is also presented to allow for comparison to a target ROI included in this study

Figure 1 displays the average of all subjects’ TACs for the cerebellum ROI, SVCA reference, and the metabolite-corrected plasma curve, as well as the thalamus ROI.

Using the 2TCM, we derived *V*_T_, *V*_S_, and BP_ND_ (*k*_3_/*k*_4_) values for the cerebellum ROIs, as well as for the SVCA4 reference TACs. ICC, AbsVar, and MD values were similar to those reported for the target ROIs (Table 2). *V*_T_ values were in the same range as described previously (see Fig. 3 in [9]).

**Table 2:** Test-retest metrics for *V*_T_, *V*_S_, and BP_ND_ values derived using the 2TCM from the cerebellum and the SVCA4 reference TACs

Method	Measure	Mean	SD	ICC	AbsVar%	SEM	MD%
Cerebellum	V_T_	0.67	0.16	0.81	13	0.07	29
	V_S_	0.43	0.11	0.67	18	0.06	39
	BP_ND_	1.91	0.31	− 0.31	21	0.36	52
SVCA4 reference TAC	V_T_	0.74	0.18	0.67	18	0.1	38
	V_S_	0.46	0.13	0.69	20	0.07	43
	BP_ND_	1.72	0.52	0.77	22	0.25	40

*ICC* intraclass correlation coefficient, *AbsVar%* percentage (of the mean) absolute variability, *SEM* standard error of the measurement, *MD%* percentage (of the mean) minimum detectable difference

### Evaluation of additional population-based kinetic classes for supervised cluster analysis

In addition to the population-based kinetic classes developed by [8], we also applied and evaluated two different sets of population-based kinetic classes for SVCA4. The first set was developed by PET researchers at VUMC [9], and the second set was developed by the Turku PET group (TPC) using the TPC (R)-[^11^C]PK11195 database which partly consists of the subjects included in this article. All test-retest metrics is presented in Table 3. The VUMC classes yielded higher average BP_ND_ values, but there were no substantial differences in reliability or precision regardless of what population-based classes were used.

**Table 3:** Test-retest metrics for BP_ND_ values from the SVCA4 method using two additional population-based kinetic classes, developed at VUMC and TPC, respectively

Method	Kinetic classes	Region	Mean	SD	ICC	AbsVar%	SEM	MD%
BP_ND _SRTM-SVCA4	TPC	FC	0.28	0.08	− 0.06	28	0.08	82
	TPC	GM	0.32	0.09	− 0.06	28	0.09	76
	TPC	HIP	0.33	0.13	− 0.16	45	0.14	117
	TPC	STR	0.25	0.16	− 0.51	84	0.19	214
	TPC	THAL	0.55	0.27	0.11	62	0.26	129
	VUMC	FC	0.43	0.12	0.02	30	0.12	74
	VUMC	GM	0.46	0.11	− 0.18	30	0.12	74
	VUMC	HIP	0.36	0.14	− 0.28	52	0.16	122
	VUMC	STR	0.45	0.13	− 0.32	37	0.14	88
	VUMC	THAL	0.6	0.16	− 0.09	27	0.16	76
BP_ND_SRTMv-SVCA4	TPC	FC	0.2	0.1	− 0.19	72	0.11	150
	TPC	GM	0.24	0.09	− 0.49	56	0.11	132
	TPC	HIP	0.25	0.07	− 0.46	39	0.09	101
	TPC	STR	0.23	0.09	0	46	0.09	105
	TPC	THAL	0.44	0.21	− 0.16	71	0.23	144
	VUMC	FC	0.37	0.1	0.21	25	0.09	67
	VUMC	GM	0.4	0.08	− 0.2	22	0.09	61
	VUMC	HIP	0.39	0.12	− 0.76	41	0.15	109
	VUMC	STR	0.46	0.31	0.02	65	0.31	184
	VUMC	THAL	0.63	0.14	0.22	24	0.12	53

*TPC* Turku PET Center, *VUMC* VU University Medical Center, *FC* frontal cortex, *GM* gray matter, *HIP* hippocampus, *STR* striatum, *THAL* thalamus, *ICC* intraclass correlation coefficient, *AbsVar%* percentage (of the mean) absolute variability, *SEM* standard error of the measurement, *MD%* percentage (of the mean) minimum detectable difference

### Convergent validity of all outcome measures

**Fig. 2:**
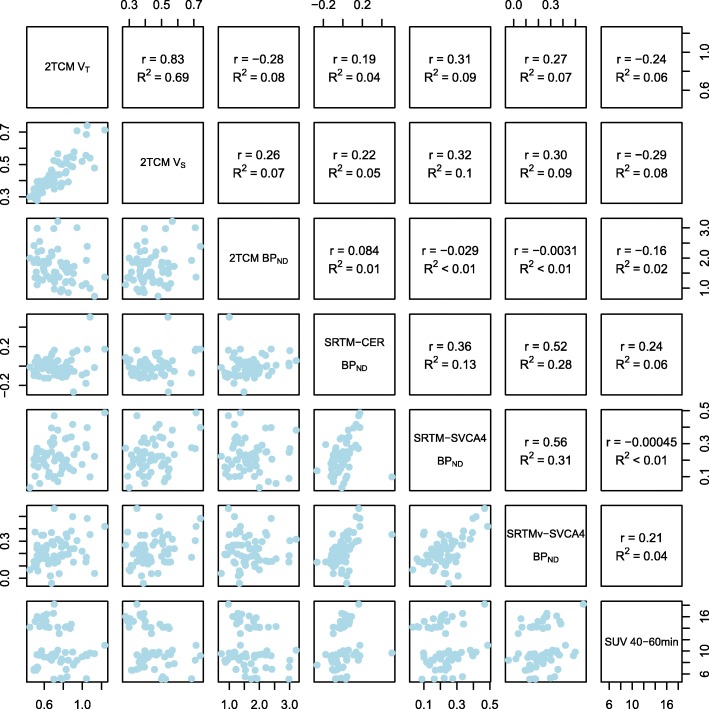
Relationships between all (R)-[^11^C]PK1195 outcome measures. Values from both PET examinations and all regions have been pooled in each panel. Pearson’s correlation coefficients (*r*) and explained variance (*R*^2^) are presented in the upper diagonal

Figure 2 shows the relationships between all (R)-[^11^C]PK1195 outcomes derived using AIF (*V*_T_, *V*_S_, and BP_ND_) and all outcomes derived without using AIF (BP_ND:SRTM-SVCA_, BP_ND:SRTMv-SVCA_, BP_ND:SRTM-CER_, and SUV). The correlation between BP_ND_ from 2TCM and BP_ND_ from SRTM-SVCA4, SRTMv-SVCA4, or SRTM-CER was negligible to non-existent, with an explained variance (R^2^) < 2% for all associations. *V*_T_ and *V*_S_ were highly correlated (R^2^ = 69%), but neither showed a strong association with BP_ND_ from AIF (R^2^ < 9%). SUVs were not correlated to any other outcome measures (R^2^ < 9%).

### Additional analyses

We also fitted the 2TCM allowing vB to differ for each ROI. Ensuing BP_ND_ (*k*_3_/*k*_4_) values showed better reliability (mean ICC = 0.6) and repeatability (mean AbsVar = 23%) compared to BP_ND_ when fitted with a fixed vB, but the test-retest metrics for *V*_T_ and *V*_S_ were notably worse (*V*_T_ mean ICC = 0.33, mean AbsVar = 21%; *V*_S_ mean ICC = 0.29, mean AbsVar = 25%, see Additional file 1: Table S1). The correlation between 2TCM *V*_T_, *V*_S_, and BP_ND_ and all other BP_ND_ outcomes (SRTM-CER, SRTM-SVCA, and SRTMv-SVCA) was still low to negligible (all *R*^2^ < 9%, see Additional file 1: Figure S1).

We also evaluated different time intervals for the SUV outcome: 10–30, 20–40, and 30–50 min. No interval yielded superior test-retest metrics (ICC values ranged from 0.80 to 0.82, AbsVar 21 to 22%) compared to 40–60 min, and correlations to remaining outcomes were still negligible to low (all *R*^2^ < 12%). All SUV intervals were however strongly correlated to each other (all *R*^2^ > 95%).

## Discussion

The objective of this study was to examine the test-retest reliability and convergent validity of (R)-[^11^C]PK11195 outcomes commonly applied in clinical in vivo studies of TSPO binding. Specifically, we evaluated outcome measures of radioligand brain exposure and binding which do not make use of an arterial input function (AIF) and compared them with binding outcomes derived using an AIF (i.e., *V*_T_, *V*_S_, and BP_ND_ from the 2TCM).

There was a striking difference in magnitude between BP_ND_ values from 2TCM using an AIF and BP_ND_ values from SRTM-SVCA4 and SRTM-CER, with much higher BP_ND_ values obtained using the 2TCM compared to the other two measures. This signifies that the use of SVCA, as well as the cerebellum, for derivation of a reference TAC yields only relative or pseudo-BP_ND_ values. TSPO is expressed throughout the brain, and specific binding is to be expected in every voxel [18, 30]. Hence, it is unlikely that SVCA4 or the cerebellum can be used to establish a TAC that reflects a true reference, devoid of TSPO, for (R)-[^11^C]PK11195.

In general, all (R)-[^11^C]PK11195 outcome measures analyzed in this study showed poor to moderate reliability. For the whole GM ROI, only SUV and *V*_T_ showed acceptable reliability (ICC > 0.65) [31]. Assuming that the true TSPO concentration is stable between PET examinations, an ICC of 0.5 suggests that as much of the variance in the sample is attributable to true signal as can be attributed to measurement error and noise. All outcomes derived without the use of an AIF showed ICC values around or below 0.5, suggesting poor reliability for these measures. SRTM with the cerebellum as reference region showed the largest imprecision and MD. These results suggest that a change in BP_ND_ from SRTM_CER_ would need to be, on average, larger than 10 times the mean in order to detect a true difference between two measurements of the same subject. In comparison, a change in *V*_S_ of (on average) 40% would be necessary to detect a difference that is not only due to noise. The lack of reliability and precision for BP_ND_ from the cerebellum and SVCA can likely be explained by having a similar (low) specific to non-specific binding ratio in both the target and pseudo-reference regions, leading to TACs which are similar in both shape and magnitude. This yields BP_ND_ values which are close to zero (or negative) and which are sensitive to even small amounts of measurement error.

Another limitation when using the cerebellum as reference is that it requires researchers to establish significant *equivalence* [32, 33] in reference region-specific binding between the groups which are being compared. A non-significant difference between groups does not translate into evidence in favor of an absence of a difference [34], contrary to conclusions sometimes drawn in literature.

*V*_T_, *V*_S_, and BP_ND_ derived from 2TCM showed little to no correlation with BP_ND_ derived using outcomes without an AIF. This indicates that BP_ND_ from the reference input models have little to no convergent validity in relation to binding outcomes from AIF, and vice versa. Hence, if either *V*_T_, *V*_S_, or BP_ND_ derived using an AIF is to be considered to be at least moderately associated with specific TSPO binding, then BP_ND_ derived without the use of AIF cannot be considered valid. However, BP_ND_ from AIF also produced low ICC values and a negligible association with *V*_T_ and *V*_S_, suggesting that this outcome is also unreliable and unstable. SUVs showed the highest average reliability but were not correlated with any other outcome measures.

In healthy control subjects, a large portion of the (R)-[^11^C]PK11195 signal consists of non-specific binding and unbound radioligand, as determined by blocking studies showing BP_ND_ values in the range of 0.8–0.9 [35]. As described, a low signal for specific binding in healthy controls may partly explain the low reliability observed in this study. In comparison, much higher reliability has been shown for SVCA in patients with Alzheimer’s disease [8] where glial cell markers are known to be elevated based on postmortem studies [36]. This gains partial support from the fact that second generation TSPO tracers, which show higher specific binding [37], also display higher ICC values in healthy control subjects [38]. It is hence likely that (R)-[11C]PK11195 outcomes would show higher reliability in clinical populations with a significant increase in brain TSPO. However, this would also imply that differences between patients and controls would need to be very large in order to be detectable. While such effects may be present in some patient groups, such as patients with stroke [39], caution is advised for disorders for which increases in TSPO might be more subtle.

One caveat with this study is that the kinetic classes used for the SVCA method are sensitive to differences in the PET system, such as system type and acquisition protocol. It cannot be excluded that other scanners might have shown higher test-retest values for the SVCA outcomes, using the same design. Importantly, the 6-week interval between PET measurements in this study means that TSPO levels may change from test to retest. This, in turn, would lead to lower reliability and precision. However, since many clinical studies aim to evaluate longitudinal interventions or correlate (R)-[^11^C]PK11195 outcomes with more stable independent variables, this interval mimics that of realistic and relevant designs of PET studies. In addition, the time between measurements also should not impact the relative reliability between different outcome measures of specific binding (such as *V*_S_ and BP_ND_), nor does it affect the evaluation of convergent validity.

## Conclusions

The results from this study suggest that caution is warranted for the application and interpretation of (R)-[^11^C]PK11195 BP_ND_ obtained using 2TCM or BP_ND_ from kinetic models using the cerebellum or SVCA4 as reference. *V*_T_ and *V*_S_ should likely be preferred over BP_ND_ from 2TCM, since they exhibited higher reliability and precision. However, the negligible correlations of *V*_T_ and *V*_S_ to SUVs are concerning and not fully understood. One explanation might be that brain SUV values are sensitive to changes in peripheral binding of TSPO [7], while AIF-based outcomes are not. This hypothesis warrants further investigation in future studies.

## Additional file

Additional file 1: **Figure S1.** Relationships between all (R)-[11C]PK1195 outcome measures, where whole-blood contribution to ROI radioactivity (vB) has been fitted for each ROI. Values from both PET examinations and all regions have been pooled in each panel. Pearson’s correlation coefficients (*r)* and explained variance (*R*^2^) are presented in the upper diagonal. **Table S1.** Test-retest metrics for BPND, *V*_S_, and *V*_T_ values derived using 2TCM while fitting vB for each ROI separately. (PDF 157 kb)

## Abbreviations

2TCM: Two-tissue compartment model
AbsVar: Absolute variability (or test-retest variability)
AIF: Arterial input function
BP_ND_: Non-displaceable binding potential
CER: Cerebellum
FC: Frontal cortex
GM: Gray matter
HIP: Hippocampus
ICC: Intra-class correlation coefficient
MD: Minimum detectable difference
MRI: Magnetic resonance imaging
PET: Positron emission tomography
ROI: Region of interest
SEM: Standard error of measurement
SRTM: Simplified reference tissue model
SRTMv: Simplified reference tissue model with additional modeling of vB
STR: Striatum
SUV: Standardized uptake values
SVCA: Supervised cluster analysis
TAC: Time-activity curve
THA: Thalamus
TSPO: Translocator protein 18 kDa
vB: Radioactivity contribution from whole blood
*V*_S_: Specific distribution volume
*V*_T_: Total distribution volume
