# Supplementary material for: Test-retest reliability and convergent validity of (R)-[11C]PK11195 outcome measures without arterial input function
Source: EJNMMI Res. 2018 Nov 29;8:102. doi: 10.1186/s13550-018-0455-8 (PMC6265355; doi:10.1186/s13550-018-0455-8)
Supplement: Supplementary file 1 — Figure S1. Relationships between all (R)-[11C]PK1195 outcome measures, where whole-blood contribution to ROI radioactivity (vB) has been fitted for each ROI. Values from both PET examinations and all regions have been pooled in each panel. Pearson’s correlation coefficients (r) and explained variance (R2) are presented in the upper diagonal. Table S1. Test-retest metrics for BPND, VS, and VT values derived using 2TCM while fitting vB for each ROI separately. (PDF 157 kb) [file 13550_2018_455_MOESM1_ESM.pdf]

Supplementary Material

Plavén-Sigray et al.

Evaluation of 2TCM outcomes with vB fitted for each ROI

We also evaluated the reliability and convergent validity of 2TCM outcome measures, were vB had been fitted separately for each subject and and ROI. 2TCM BP_{ND} showed on average higher reliability and repeatability, compared to when fitting one vB for the entire brain (Supplementary Table 1). However, 2TCM V_S and V_T showed notably lower test-retest values with a vB fitted for each ROI. There were no to low correlations to remaining outcomes (BP_{ND} from SRTM-CER, SRTM-SVCA, SRTMv-SVCA or SUV40-60 minutes, see Supplementary Figure 1).

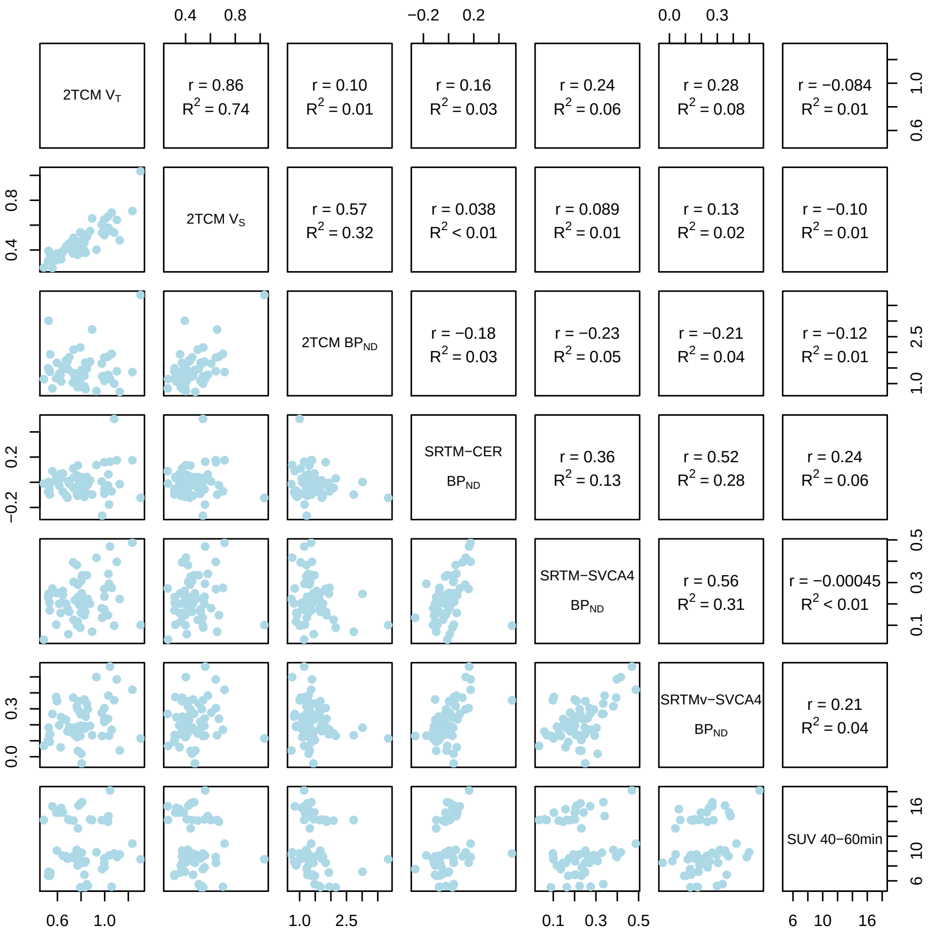

Figure 1: Relationships between all (R)-[^{11}C]PK1195 outcome measures, where whole-blood contribution to ROI radioactivity (vB) have been fitted for each ROI. Values from both PET examinations and all regions have been pooled in each panel. Pearson's correlation coefficients (r) and explained variance (R^2) are presented in the upper diagonal.

Table 1: Test-retest metrics for BPND, VS and VT values derived using 2TCM while fitting vB for each ROI separately.

Region	Mean	SD	ICC	AbsVar%	SEM	MD%
**BPND**						
FC	1.42	0.35	0.82	13	0.15	29
GM	1.34	0.29	0.76	13	0.14	29
HIP	1.70	0.92	0.74	35	0.46	76
STR	1.46	0.49	0.11	30	0.46	87
THAL	1.30	0.33	0.57	24	0.22	46
**VS**						
FC	0.46	0.10	0.54	16	0.07	39
GM	0.45	0.10	0.59	17	0.06	38
HIP	0.49	0.20	0.03	34	0.20	113
STR	0.47	0.12	-0.02	31	0.12	70
THAL	0.46	0.14	0.33	27	0.11	66
**VT**						
FC	0.80	0.15	0.51	15	0.10	36
GM	0.79	0.16	0.59	16	0.10	36
HIP	0.81	0.24	-0.01	26	0.24	81
STR	0.80	0.16	0.22	23	0.14	49
THAL	0.84	0.23	0.33	27	0.19	63
